# Supplementary figures and images for: Transient Superdiffusion and Long-Range Correlations in the Motility Patterns of Trypanosomatid Flagellate Protozoa
Source: PLoS One. 2016 Mar 23;11(3):e0152092. doi: 10.1371/journal.pone.0152092 (PMC4805249; doi:10.1371/journal.pone.0152092)

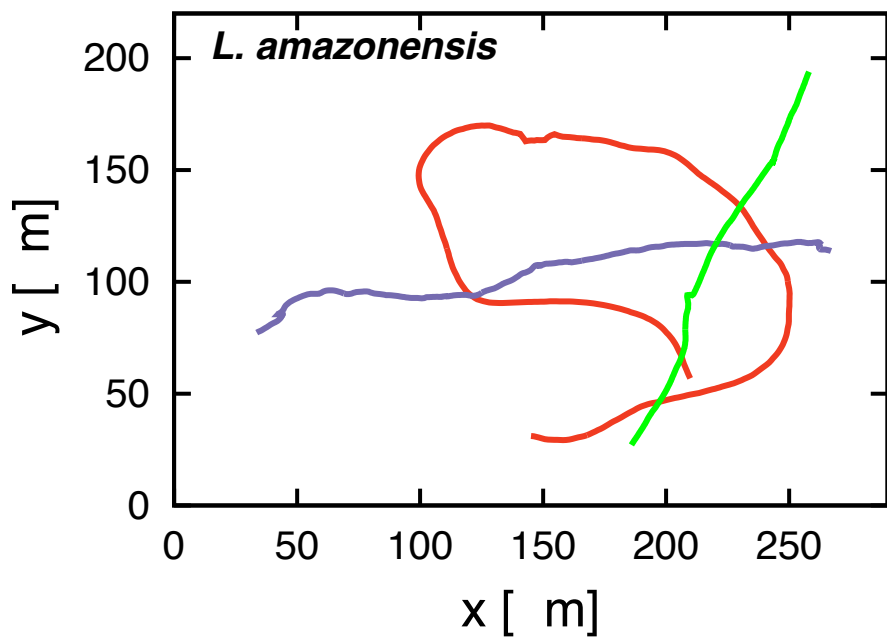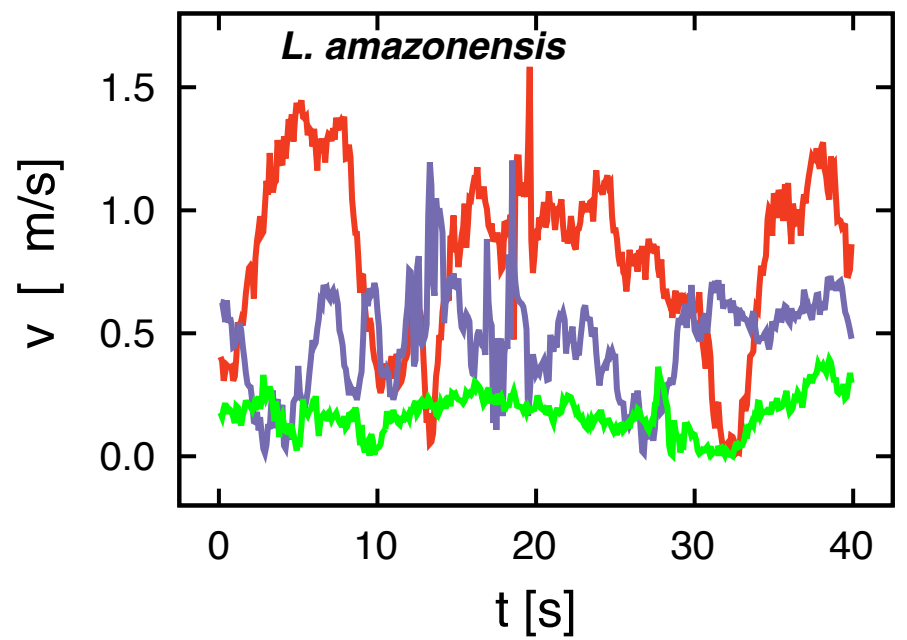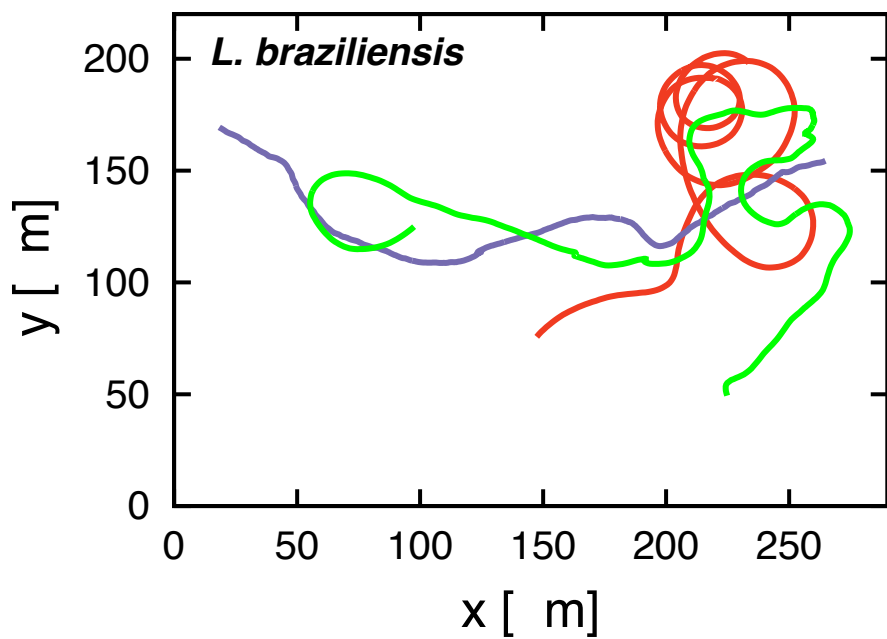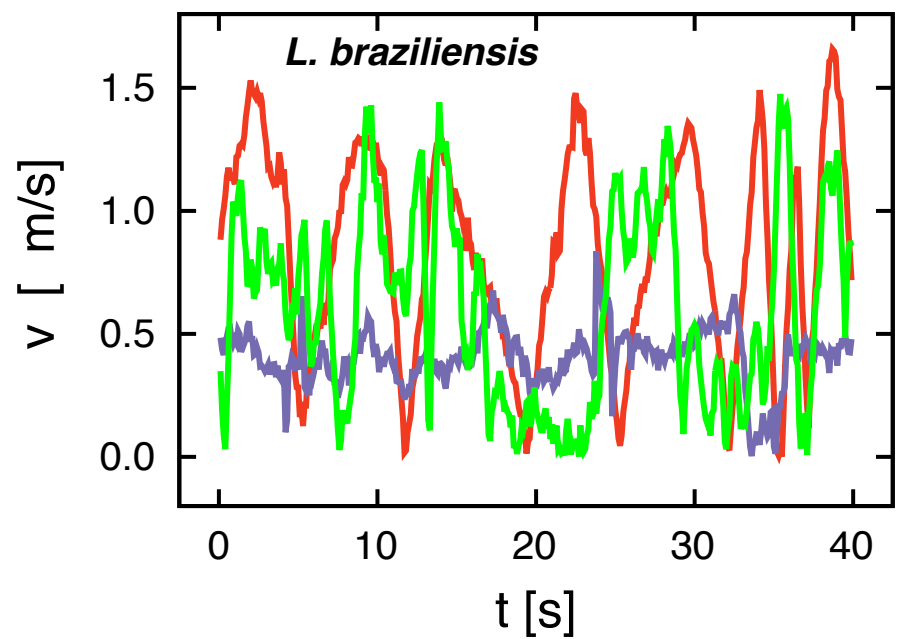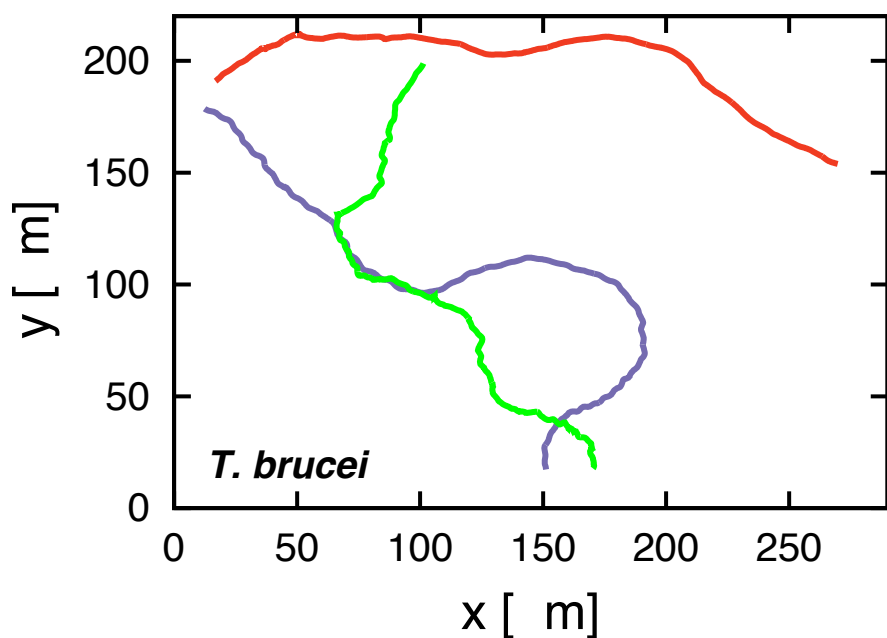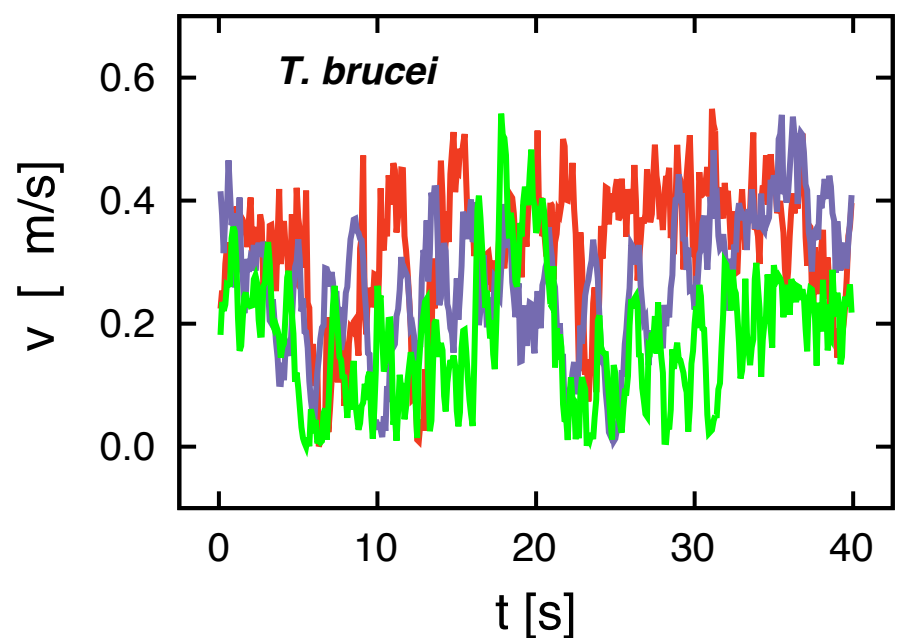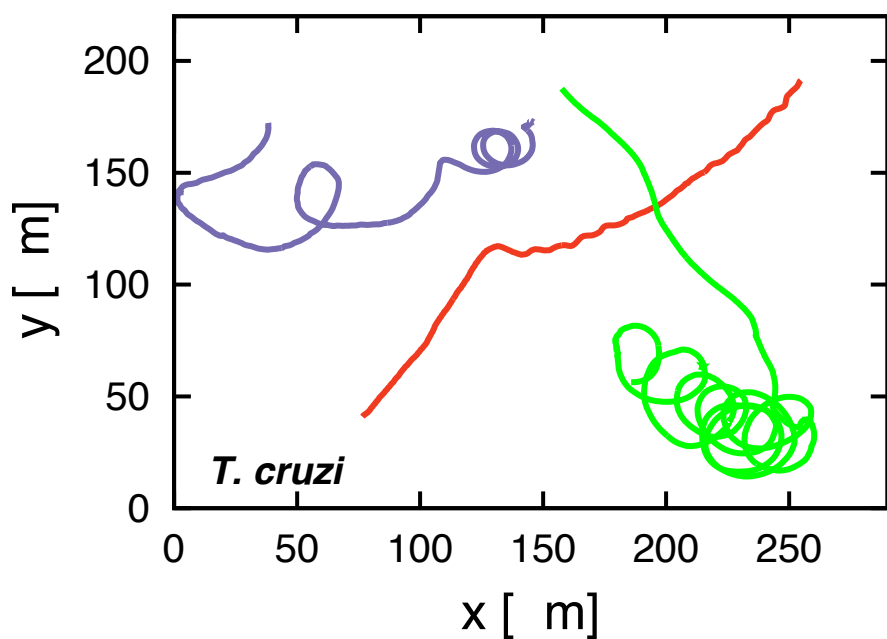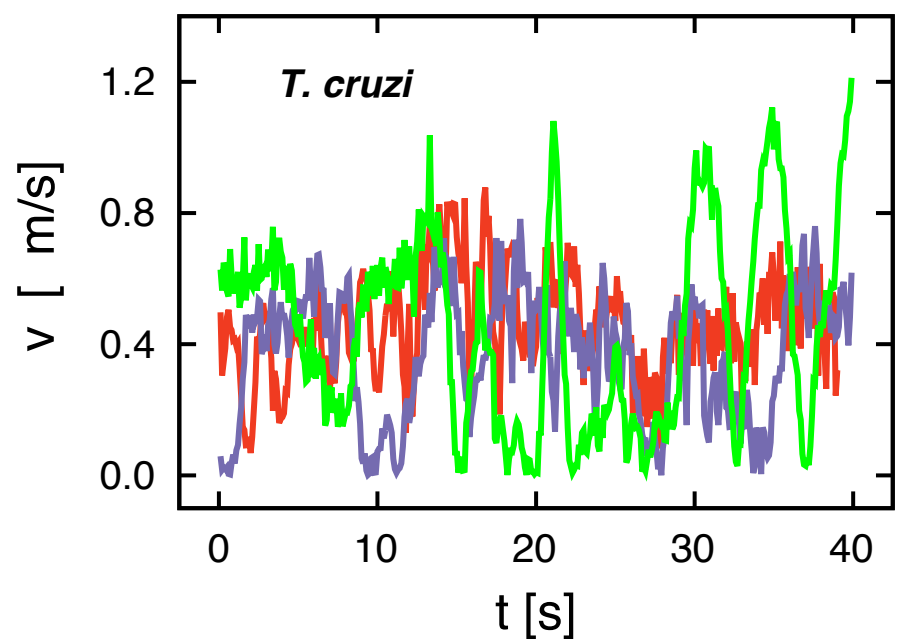

Supplement: S1 Fig — The left panel shows typical trajectories of the four protozoa. The right panel shows the corresponding velocity time series v(t). (PDF) [file pone.0152092.s002.pdf]

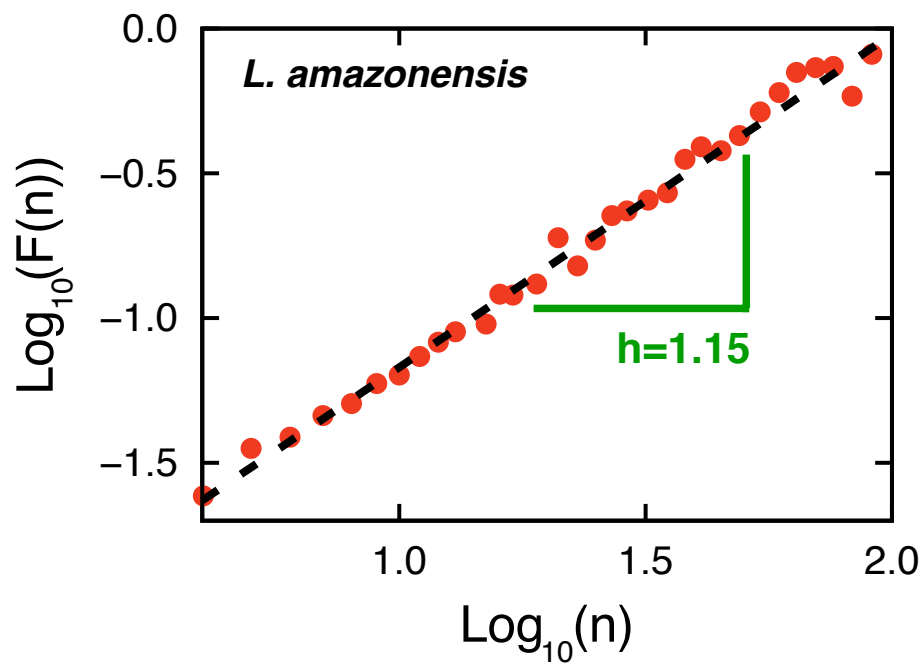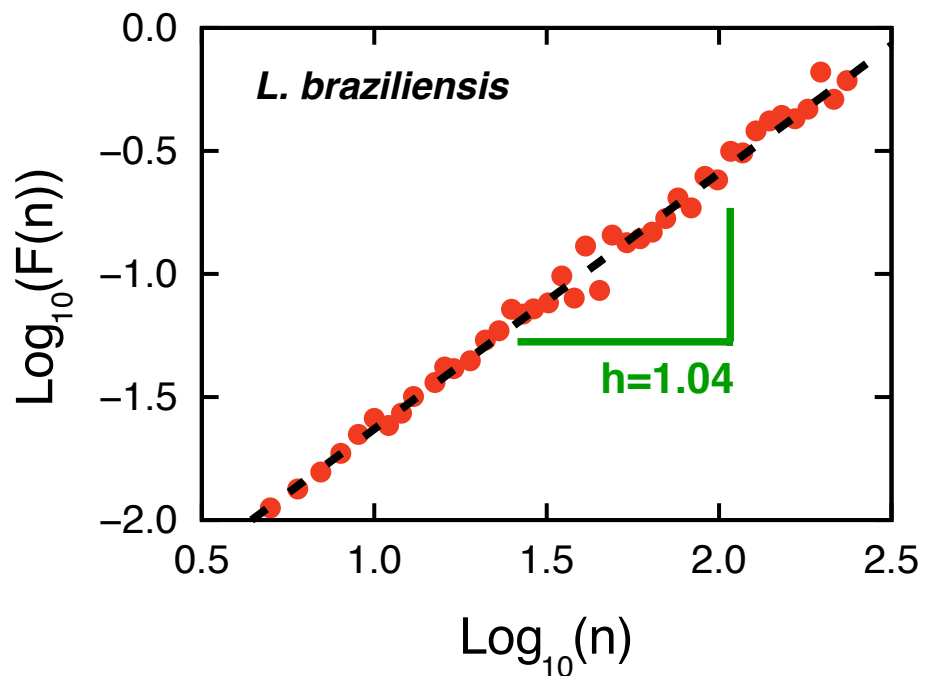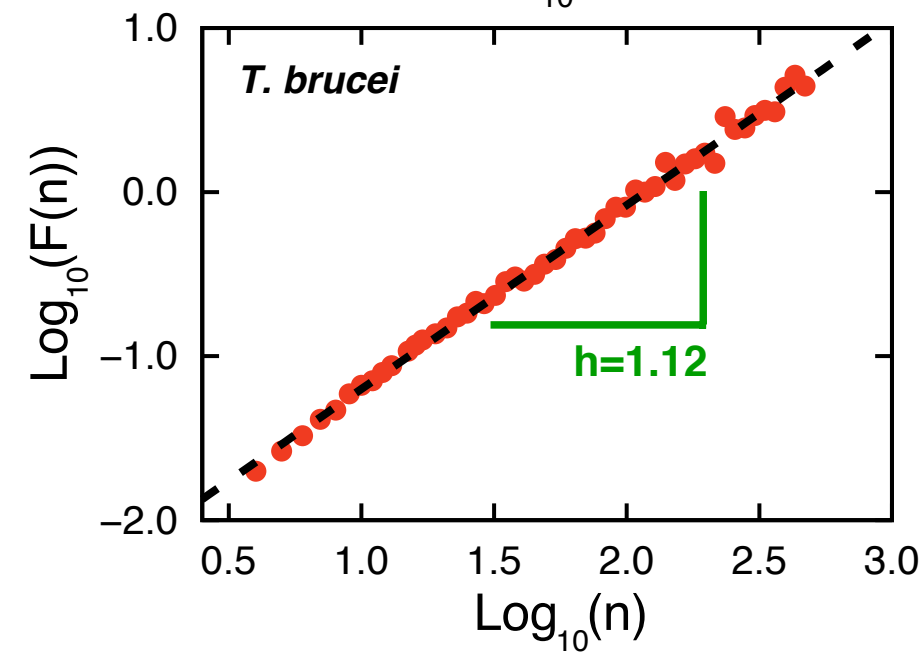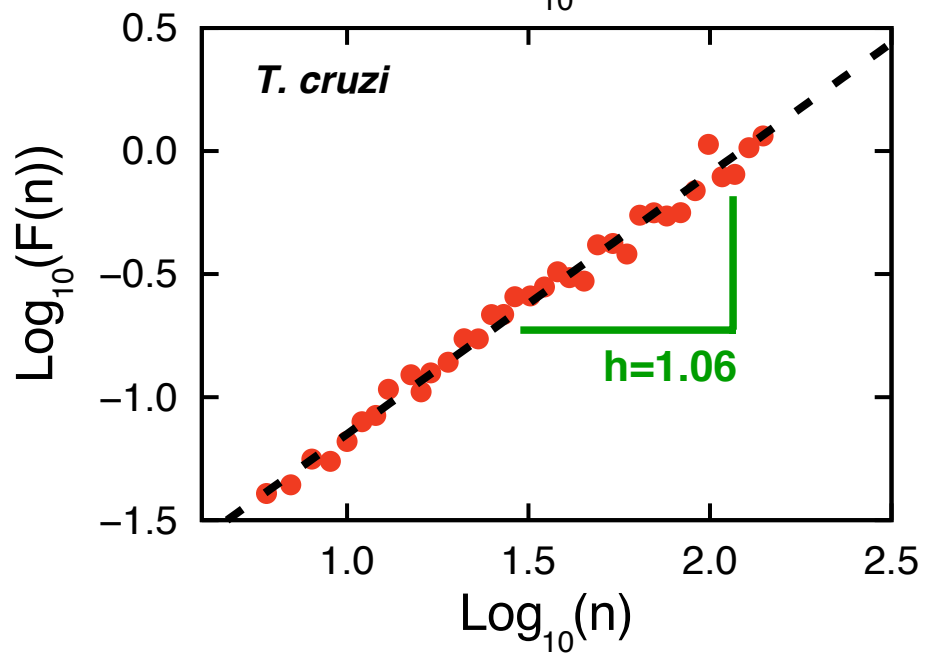

Supplement: S2 Fig — Another typical examples of detrended fluctuation analysis (DFA) for the velocity time series. (PDF) [file pone.0152092.s003.pdf]

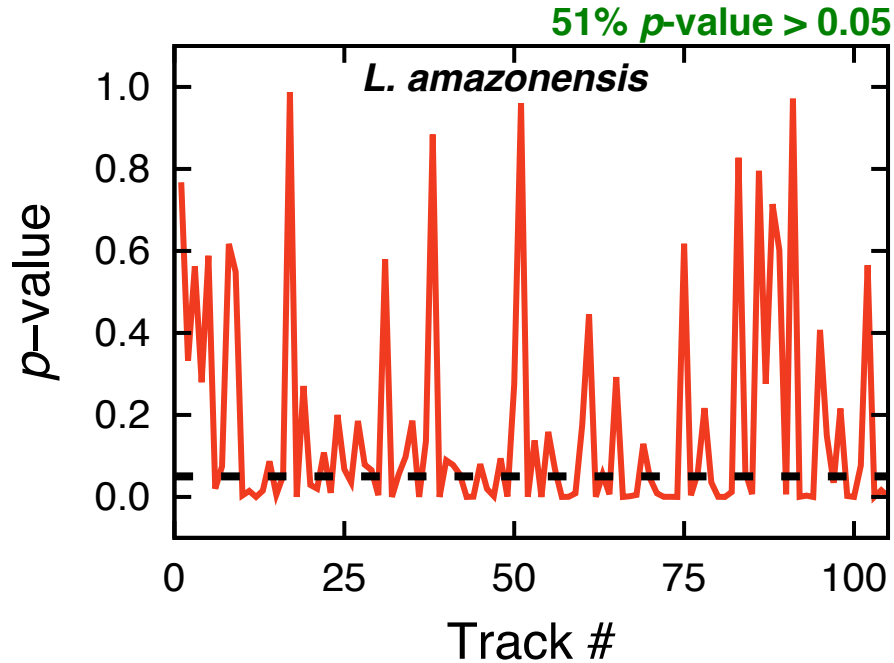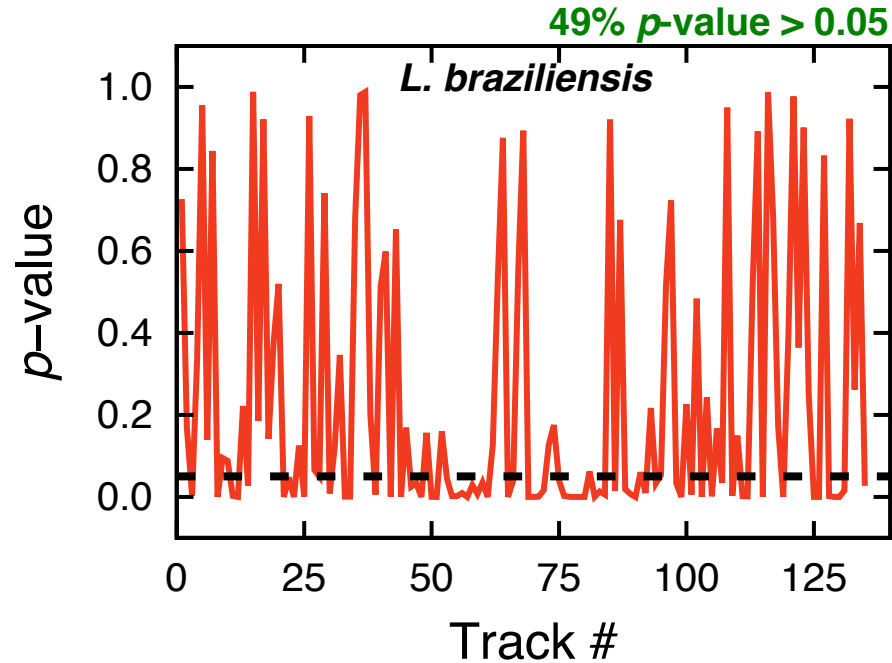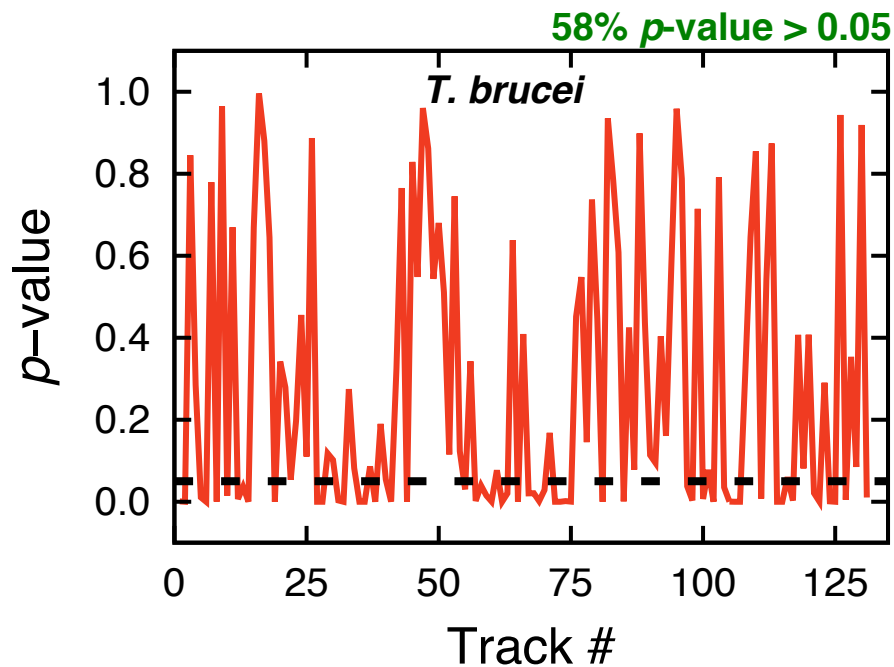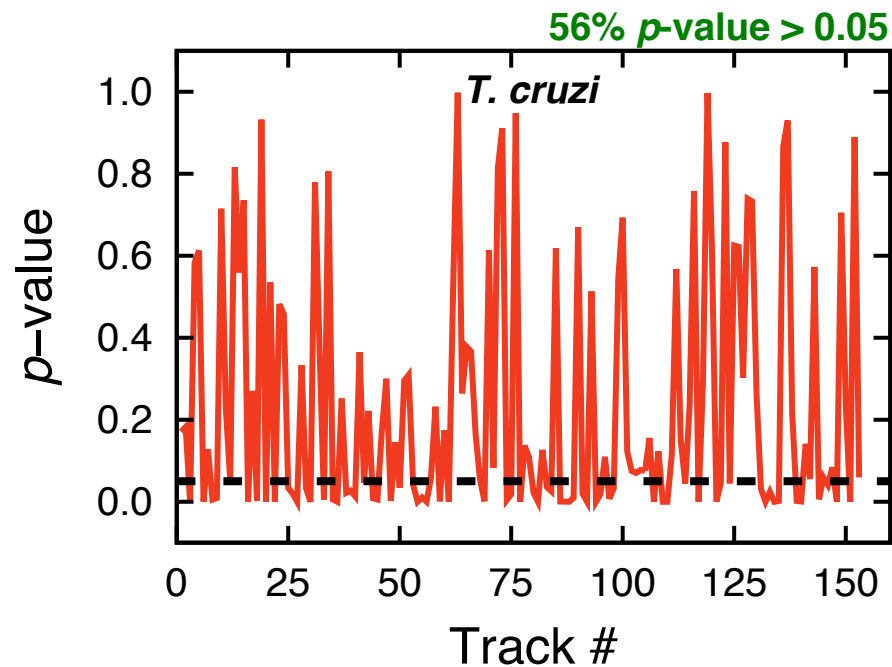

Supplement: S3 Fig — The red lines show the p-values of Kolmogorov-Smirnov test of the generalized gamma hypothesis for all velocities time series. About 50% of the velocity time series have p > 0.05 (horizontal line). (PDF) [file pone.0152092.s004.pdf]
